# Unresolved orthology and peculiar coding sequence properties of lamprey genes: the *KCNA *gene family as test case

**DOI:** 10.1186/1471-2164-12-325

**Published:** 2011-06-23

**Authors:** Huan Qiu, Falk Hildebrand, Shigehiro Kuraku, Axel Meyer

**Affiliations:** 1 Laboratory for Zoology and Evolutionary Biology, Department of Biology, University of Konstanz, Universitätsstrasse 10, 78457 Konstanz, Germany; 2 Konstanz Research School Chemical Biology (KoRS-CB), University of Konstanz, Universitätsstrasse 10, 78457 Konstanz, Germany; 3 Bigelow Laboratory for Ocean Sciences, 180 McKown Point Road, West Boothbay Harbor, Maine 04575-0475, USA; 4 Bioinformatics and (eco-) systems biology, VIB, Vrije Universiteit Brussel, 1050 Brussels, Belgium

## Abstract

**Background:**

In understanding the evolutionary process of vertebrates, cyclostomes (hagfishes and lamprey) occupy crucial positions. Resolving molecular phylogenetic relationships of cyclostome genes with gnathostomes (jawed vertebrates) genes is indispensable in deciphering both the species tree and gene trees. However, molecular phylogenetic analyses, especially those including lamprey genes, have produced highly discordant results between gene families. To efficiently scrutinize this problem using partial genome assemblies of early vertebrates, we focused on the potassium voltage-gated channel, shaker-related (*KCNA*) family, whose members are mostly single-exon.

**Results:**

Seven sea lamprey *KCNA *genes as well as six elephant shark genes were identified, and their orthologies to bony vertebrate subgroups were assessed. In contrast to robustly supported orthology of the elephant shark genes to gnathostome subgroups, clear orthology of any sea lamprey gene could not be established. Notably, sea lamprey *KCNA *sequences displayed unique codon usage pattern and amino acid composition, probably associated with exceptionally high GC-content in their coding regions. This lamprey-specific property of coding sequences was also observed generally for genes outside this gene family.

**Conclusions:**

Our results suggest that secondary modifications of sequence properties unique to the lamprey lineage may be one of the factors preventing robust orthology assessments of lamprey genes, which deserves further genome-wide validation. The lamprey lineage-specific alteration of protein-coding sequence properties needs to be taken into consideration in tackling the key questions about early vertebrate evolution.

## Background

For a complete understanding of the evolution of vertebrates, jawless fishes (cyclostomes; hagfishes and lampreys) occupy crucial positions as the most early-branching lineages, which diverged more than 500 million years ago from the gnathostome (jawed vertebrate) lineage [1]. Their phylogenetic relationships with gnathostomes serve as a crucial scaffold on which one can map phenotypic and genotypic changes [2,3]. More importantly, genome expansions, known as 'two-round whole genome duplications (2R-WGDs)', took place around the divergences of these lineages [4,5].

**Figure 1:**
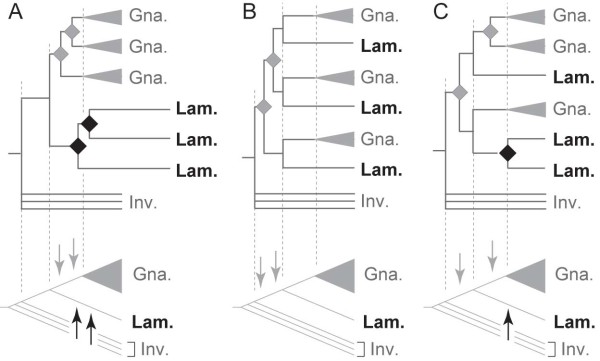
**Alternative tree toplogies supporting different timings of two-round whole genome duplications (2R-WGDs)**. As an example, for a gene family with three gnathostome paralogs and three lamprey paralogs, gene trees (top) and species trees with timings of gene duplications (bottom) are shown. Gene duplications that gave rise to multiple gnathostome paralogs are indicated with grey diamond (top) and grey arrows (bottom), and those in the lamprey lineage are indicated with black diamond (top) and black arrows (bottom). Even though a recent large-scale phylogenetic analysis supported the scenario in B [4], analyses on single gene families often result in the tree topology similar to that in A. C was previously supported [49]. Abbreviations: Gna., gnathostome gene; Lam., lamprey gene; Inv., invertebrate gene.

It has recently been shown that the 2R-WGDs occurred in the stem lineage leading to vertebrates after the splits of the cephalochordate and urochordate lineages [6] before the chondrichthyan lineage branched off [7]. Recently, a scenario in which both WGDs occurred before the cyclostome-gnathostome split was suggested [4]. However, there are many gene families that do not conform to the expected tree topology in phylogeny reconstructions. Often phylogenetic studies recover basal divergences and exclusive groupings of lamprey sequences with long branches (Figure 1A; see refs [8-14] for examples). This feature, observed commonly among different gene families, has also been interpreted as in support of lamprey lineage-specific genome duplication [15]. It would appear that methodological biases add to the difficulty in reconstructing the molecular phylogenies, originating from possible peculiar features of lamprey sequences, which might be preventing proper reconstructions of gene family trees.

**Figure 2:**
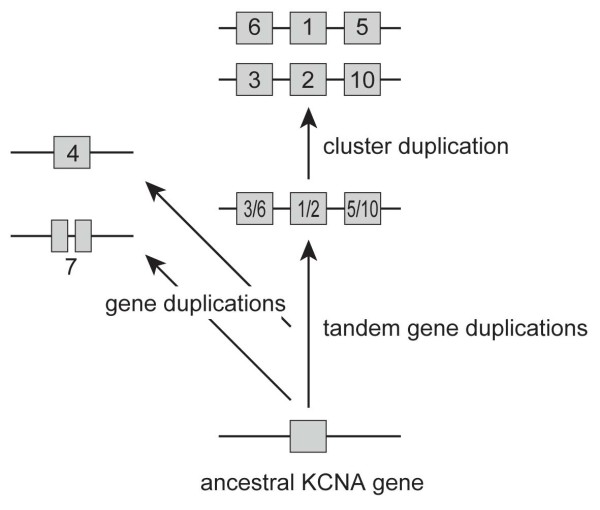
**Proposed scenario for the evolution of *KCNA *genes and clusters in vertebrates**. Names of genes are indicated inside or below boxes showing *KCNA *genes. In the human genome, there are eight *KCNA *genes, six of which form two tri-gene clusters (*KCNA6-1-5 *and *KCNA3-2-10*). The human *KCNA *clusters are positioned on chromosomes 1 (*KCNA6-1-5*) and 12 (*3-2-10*). Except for *KCNA7*, which has one intron, all vertebrate *KCNA *genes identified so far are intronless. The status with a single tri-gene cluster is hypothetical.

To reveal possible peculiar features of lamprey protein-coding sequences, we focused on the potassium voltage-gated channel, shaker-related subfamily (*KCNA*) [16]. Members of this gene family are mostly single-exon genes. Thus, they are particularly ideal candidate genes for this type of analysis involving partial genome sequences that are available for the sea lamprey (*Petromyzon marinus*) and the elephant shark (*Callorhinchus milii*). In tetrapods, the *KCNA *family comprises eight genes, six of which form two tri-gene clusters that were established by tandem duplications followed by a chromosomal duplication (Figure 2) [17]. This chromosomal duplication is thought to be part of the 2R-WGDs [17]. It is of interest to assess orthology of lamprey genes based on the available genome assembly in this already established evolutionary framework of the *KCNA *gene family.

In this study, we identified *KCNA *genes from partial genome sequences of the sea lamprey and the elephant shark, and investigated their phylogeny and sequence properties. We observed exclusive clustering of lamprey sequences with long branches as in Figure 1A. The supported tree topology did not fit any of possible scenarios supported by the evolutionary pattern of the *KCNA *gene family documented previously. Notably, our analyses on *KCNA *and other genes revealed peculiar codon usage bias and amino acid composition unique to the sea lamprey.

## Results

### Identification of sea lamprey and elephant shark *KCNA *genes

Seven full-length or nearly full-length KCNA protein-coding sequences were identified in the sea lamprey (*Petromyzon marinus*) whole genome assembly. We also identified six in the elephant shark (*Callorhinchus milii*), one in *Ciona savignyi *and one in purple urchin (*Strongylocentrotus purpuratus*). For *Ciona intestinalis*, in addition to one previously reported KCNA homolog (XP_002125274.1), one more KCNA protein sequence was found in Genbank (Additional file 1, Figure S1). Apart from the seven aforementioned *KCNA *genes, six and three fragments of *KCNA *genes (encoding < 150 amino acids peptides) were also discovered in the sea lamprey and elephant shark genome, respectively (data not shown). These remain partial probably because of incomplete genome sequencing, and they were therefore excluded from the subsequent analyses. Our search in NCBI dbEST, an expressed sequence tag (EST) archive, identified no sequences encoding KCNA proteins for species in Petromyzontiformes (lampreys), Myxiniformes (hagfishes) and Chondrichthyes (cartilaginous fishes).

### Molecular phylogenetic analysis: an overview

Phylogenetic trees were constructed with the maximum-likelihood (ML) method and Bayesian inference for 32 *KCNA *sequences, including all of the newly identified putative *KCNA *sequences mentioned above (Additional file 1, Figure S1). We used human homologs of closely related potassium channel subfamilies as an outgroup. The sea lamprey *PmKCNAβ *gene was not included because it was partial and thus largely reduced the number of aligned sites available for tree construction. Although the best tree topologies produced by these two methods differed to some extent, all the identified sequences appeared to be genuine *KCNA *homologs as they formed a cluster with already reported *KCNA *genes with high statistical support [bootstrap probability (BP): 89; posterior probability (PP): 1.00; Additional file 2, Figure S2].

**Figure 3:**
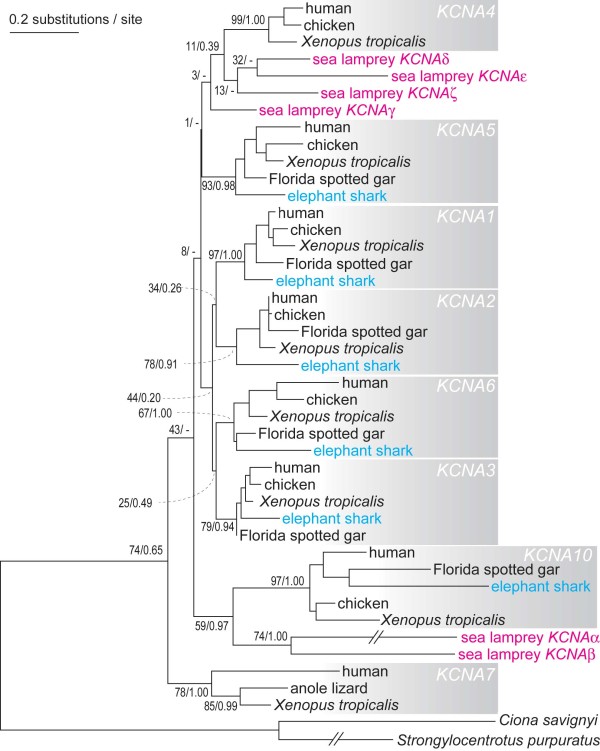
**Maximum-likelihood tree of *KCNA *genes rooted by invertebrate sequences**. 203 amino acid sites were employed in this analysis. Statistical support values for nodes with branches leading to sea lamprey and elephant shark genes are shown with ML bootstrap values left to the slash and posterior probabilities right to the slash. "-" indicates inconsistent tree topologies between Maximum-likelihood tree and Bayesian tree.

The tree focusing on the vertebrate *KCNA *genes was reconstructed with the ML (Figure 3) and Bayesian inference methods (Additional file 3, Figure S3). This analysis included six sea lamprey *KCNA *genes and complete or nearly complete sets of *KCNA *sequences of three tetrapods (human, chicken and frog), one non-teleost actinopterygian fish (Florida gar), and one cartilaginous fish (elephant shark), and employed sea urchin and sea squirt homologs as an outgroup (Figure 3). The sea lamprey *PmKCNAν *was excluded because of its extremely divergent sequence among the deuterostome *KCNA *genes (Additional file 2, Figure S2). Maximum-likelihood and Bayesian inference supported tree topologies similar to that of a previous study, in terms of relationships between gnathostome *KCNA *subgroups [17]. In this tree, monophylies of all individual gnathostome *KCNA *subgroups (*KCNA1-7 *and *10*) were strongly supported (BP: 67-99; PP: 0.91-1.00). However, instead of grouping *KCNA5 *and *-10*, which was suggested by the previously proposed evolutionary history of the *KCNA *clusters [17], a sister group relationship between *KCNA10 *and *KCNA1-6 *was supported by the ML tree (Figure 3). This might be due to the accelerated evolution of *KCNA10 *after the split between *KCNA5 *and *-10 *[17] and shorter alignment [203 amino acid sites (aa)] used in this analysis than in the previous one (364 aa) [17].

All elephant shark *KCNA *genes were relatively robustly placed in the phylogenetic trees (Figure 3), in accordance with its phylogenetic position in the species tree. In contrast, none of the sea lamprey *KCNA *genes was unambiguously assigned to any of the jawed vertebrate *KCNA *subgroups (Figure 3). Although sister relationships between *PmKCNAα/β *and *KCNA10 *as well as between *PmKCNAδ/ε/ζ *and *KCNA4 *were recovered by both methods, statistical support for these relationships were not high (BP: 11~59; PP: 0.39~0.97). Maximum-likelihood and Bayesian inference methods with different models, e.g, JTT+Γ_4_, JTT+I+Γ_4_+F and LG+I+Γ_4_, LG+I+Γ_4_+F produced similar results (data not shown).

#### Timings of gene duplications in the *KCNA *gene family

**Figure 4:**
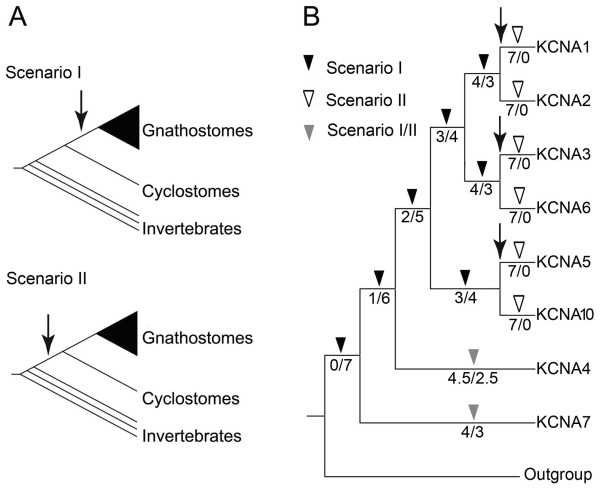
**Alternative scenarios for the timings of gene duplications in the *KCNA *family**. (A) The timing of whole genome duplication relative to cyclostome-gnathostome split. Timings of genome duplications are shown as black arrows in the species phylogeny. (B) One possible gnathostome *KCNA *phylogeny. The 15 arrowheads above the branches of the gnathostome *KCNA *phylogeny indicate possible positions of individual sea lamprey genes in the tree topology. Black arrows indicate whole genome duplication. Two alternative tree topologies connecting 1) *KCNA1/2 *and *KCNA5/10 *and *2*) *KCNA3/6 *and *KCNA5/10 *are not shown. Thus there are 45 possible tree topologies in total for a dataset containing the complete gnathostome gene set studied plus a sea lamprey gene. Arrowheads in black and white support scenarios I and II, respectively. Positioning of sea lamprey genes indicated with grey arrowheads cannot distinguish between scenario I and II. Numbers below each branch show the expected number of gene duplications for that branch before (left to the slash) and after (right to the slash) the cyclostome-gnathostome split.

The timing of *KCNA *cluster duplication relative to the WGDs was investigated by comparing the probabilities of scenario I and II (Figure 4), and probabilistic counts of gene duplications (see Methods). Of the seven sea lamprey *KCNA *genes, three (*PmKCNAα, -β *and *-ν*) had higher probabilities for scenario II than for scenario I. One gene (*PmKCNAζ*) produced comparable probabilities between scenario I and II, and the remaining three genes (*PmKCNAγ, -δ *and *-ε*) had higher probabilities for scenario I (Table 1). This approach did not provide unequivocal support for either of scenario I or II. Probabilistic counts of gene duplications (see Methods) resulted in *N*_bef _ranged from 2.9 to 6.8 and *N*_aft _from 0.2 to 4.0. Except for *PmKCNAγ, N*_bef _showed higher values than *N*_aft _for all the sea lamprey *KCNA *genes surveyed (Table 1).

**Table 1:** Probabilities of scenarios I and II and probabilistic counts of gene duplications before and after the cyclostome-gnathostome split

Gene^1^	Probabilities of hypotheses^2^		Total count of gene duplications^2^	
	
	I	II	*N*_bef_	*N*_aft_
*PmKCNAα*	0.11	0.89	6.80	0.22
*PmKCNAβ*	0.07	0.93	6.77	0.22
*PmKCNAγ*	0.85	0.15	2.94	4.08
*PmKCNAδ*	0.61	0.39	4.17	2.85
*PmKCNAε*	0.72	0.28	4.05	2.97
*PmKCNAζ*	0.51	0.49	4.79	2.22
*PmKCNAν*	0.36	0.64	5.00	2.00

^1 ^Potassium voltage-gated channel, shaker-related gene (*KCNA*) from sea lamprey.

^2 ^See Figure 4A for details of scenarios I and II and Methods for the count of gene duplications before (*N_bef_*) and after (*N_aft_*) the cyclostome-gnathostome split.

### Amino acid composition of *KCNA *genes

**Figure 5:**
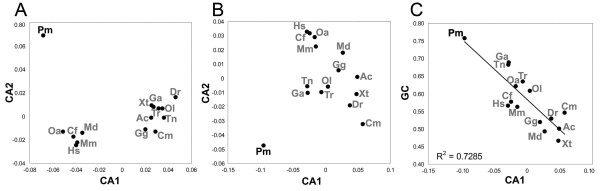
**Factor maps for amino acid compositions**. (A) amino acid composition of alignable regions of *KCNA *from 15 vertebrate species. (B) Genome-wide amino acid composition for 15 vertebrate species. (C) Correlation of the first factor of the CA of genome-wide amino acid composition with average coding GC level. CA1: the first axis of correspondence analysis. CA2: the second axis of correspondence analysis. Abbreviations for species names: Hs (*Homo sapiens*), Mm (*Mus musculus*), Cf (*Canis familiaris*), Md (*Monodelphis domestica*), Oa (*Ornithorhynchus anatinus*), Gg (*Gallus gallus*), Ac (*Anolis carolinensis*), Xt (*Xenopus tropicalis*), Dr (*Danio rerio*), Tr (*Takifugu rubripes*), Tn (*Tetraodon nigroviridis*), Ol (*Oryzias latipes*), Ga (*Gasterosteus aculeatus*), Cm (*Callorhinchus milii*), Pm (*Petromyzon marinus*).

To scrutinize sources of ambiguity in lamprey gene phylogeny, amino acid compositions in *KCNA *genes were investigated using correspondence analysis (CA) to identify factors underlying the cross-species variances among the data (Figure 5A). CA summarizes multi-dimensional variables among a dataset into a lower number of variables and thereby facilitates the identification of major sources of variations that explain most of the variance among the data. Through this approach, we identified two major axes that account for 66.9% and 22.8% of total variance of amino acid composition in reliably aligned regions, respectively. Sea lamprey resided far from the jawed vertebrates along both axes (Figure 5A). Two compact clusters were observed within gnathostomes, with the first being exclusively composed of mammals and the other of non-mammalian gnathostomes (Figure 5A). An analysis based on the full length of *KCNA *sequences with the same set of species produced similar results (data not shown).

We performed the CA of amino acid compositions in genome-wide sequences data of 15 vertebrate species (Figure 5B; see Methods for details of the dataset for the sea lamprey). The identified first two axes accounted for 63.3% and 21.6% of total variance, respectively. Whereas teleost fishes were located with tetrapods along the first axis (CA1), mammals and chicken were distinguishable from the other vertebrates along the second axis (CA2) (Figure 5B). Contrary to the elephant shark signature that was similar to those of osteichthyes, the sea lamprey was located at an extremely distant position away from the cluster of other species along both axes (Figure 5B). GC-content has been shown to be the major determinant of amino acid composition and codon usage bias, for example, in bacteria [18] and nematodes [19,20]. Thus, we plotted the first factor of CA of amino acid composition (CA1) against the average GC-content of third positions in protein-coding regions (GC_3_) of the corresponding coding nucleotide sequence data (Figure 5C). A strong correlation was observed between these two factors (Figure 5C).

### Synonymous codon usage of *KCNA *genes

**Figure 6:**
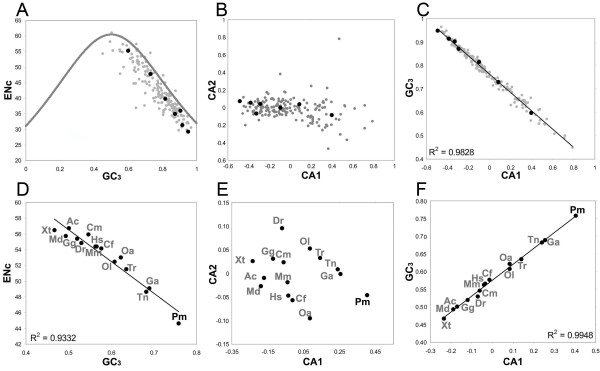
**Comparison of codon usage for *KCNA *and other genes**. (A) Plot of ENc against GC_3 _of sea lamprey genes. The curve indicates expected ENc-GC_3 _pattern under the assumption that there is no selection acting on codon usage. *KCNA *genes are shown as black dots and the remaining gene are represented by grey dots. (B) Factor map for synonymous codon usage of sea lamprey genes by crossing the first and second axes of WCA. *KCNA *genes are shown as black dots and the remaining gene are represented by grey dots. (C) Plot of the first factor of the WCA of codon usage in the sea lamprey against GC_3 _values of sea lamprey gene. *KCNA *genes are shown as black dots and the remaining gene are represented by grey dots. (D) Plot of genome-wide ENc against GC_3 _of 15 vertebrate species. (E) Factor map for synonymous codon usage of 15 vertebrate species at genome-wide level. (F) Plot of the first factor of the WCA of genome-wide synonymous codon usage against genome-wide GC_3_. Species names are abbreviated as in Figure 5.

We also focused on possible peculiarity in nucleotide sequences of the lamprey. The overall codon usage bias of each sea lamprey gene was estimated with Effective number of codons index (ENc) [21] as measure (see Methods). The higher ENc values represent greater degree of deviation from the assumption of unbiased codon usage. While sea lamprey genes showed a broad range of ENc, most genes had GC_3 _that ranged from 50% to 90% (Figure 6A). The plot of ENc and GC_3 _showed that the distribution of ENc values was close to the expected values, if no translational selection (or other biases) are acting upon the sequence (Figure 6A). In the factor map crossing the first two axes (accounting for 31% and 7% of total variance, respectively) of within-group correspondence analysis (WCA) of codon usage [22,23], lamprey genes generally displayed a uniform pattern of codon usage (Figure 6B). The first factor of the within-group correspondence analysis (CA1) was strongly correlated with GC_3 _values of sea lamprey genes (Figure 6C). No correlation was observed between the second factor of WCA and GC_3 _(data not shown). In all of the above plots, the seven sea lamprey *KCNA *genes were broadly scattered within the distribution of all genes, indicating that there were no special features unique to the *KCNA *gene family.

**Figure 7:**
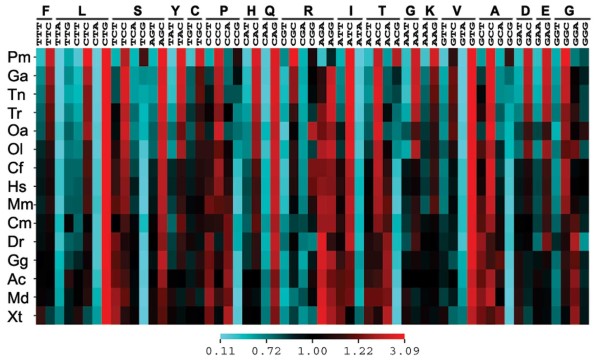
**Heat map of RSCU values for 15 vertebrate species**. Each column represents a codon, and each row represents a species. Sequence datasets employed are the same as those in Figure 6D-F. Frequencies of each synonymous codon are indicated by the colour in the relevant grid. Species are sorted according to their genome-wide GC_3 _values, and their names are abbreviated as in Figure 5.

To compare codon usage biases across species, the ENc was calculated using the available sea lamprey coding sequence dataset as well as 14 other representative vertebrates (see Methods). The lowest value of ENc (indicating the most pronounced codon usage bias) was observed for sea lamprey (ENc: 42.41), followed by stickleback (ENc: 48.62) and *Tetraodon nigroviridis *(ENc: 49.11). The ENc values were highly correlated with genome-wide average coding nucleotide GC levels (Figure 6D). The genome-wide GC_3 _is also strongly correlated with average coding GC levels across the species studied (R^2 ^= = = 96.4%, data not shown). To compare the codon usage pattern of the sea lamprey with those of other vertebrate species, correspondence analysis of codon usage was performed. In the factorial map of the WCA of codon usage, with the first two axes accounting for 84% and 8% of total variance respectively, the sea lamprey was clearly located apart from other vertebrates along the first axis, but not second axis (Figure 6E). A strong correlation was found between the first factor of CA and GC_3 _values in all vertebrates surveyed here (Figure 6F). Correspondence analysis of relative synonymous codon usage (CA-RSCU) generated similar results to WCA (data not shown). Furthermore, the overall codon usage in the available sea lamprey coding sequence dataset and genome-wide coding nucleotide sequences for other species were tabulated (Figure 7). Sea lamprey displayed a similar pattern of preference in codon usage to those of other species. However, for most amino acids it showed either the highest or the lowest frequencies for the preferred and non-preferred codons. Overall, the sea lamprey tends to have higher frequencies for GC-rich codons and lower frequencies for GC-poor ones compared to other species (Figure 7).

## Discussion

### Gene repertoires in early vertebrates

Although the genome assemblies for sea lamprey and elephant shark are incomplete, the numbers of *KCNA *genes identified in the present study (seven for the sea lamprey and six for the elephant shark) are close to the number of *KCNA *paralogs in bony vertebrates (eight in non-teleost fishes). Previously, Grus and Zhang assessed the coverage of the available sea lamprey genome assembly, and suggested that at least one exon per multi-exon gene should be identifiable in the current lamprey genome assembly [24]. However, the possibility of missing a *KCNA *gene cannot be ruled out given the single-exon nature of members of this gene family. Since the elephant shark genome was so far sequenced only with 1.4-fold coverage, more genes, such as *KCNA4 *and *-7*, may still remain to be discovered.

### Timing of whole genome duplications

We observed the ambiguity of the phylogenetic positions of the sea lamprey *KCNA *genes in conventional best tree searches (Figure 3) as well as in the likelihood-based tree topology tests that resulted in multiple tree topologies with similar likelihood values (Additional file 4, Table S1). In terms of the timing of the duplication of two *KCNA *clusters, phylogeny-based probabilistic analysis did not provide strong evidence, although probabilistic counts of individual gene duplications slightly favored the scenario with the cluster duplication before the cyclostome-gnathostome split (Table 1). The currently available sea lamprey genome assembly does not contain any intact *KCNA *gene cluster, probably because of its fragmental nature. If lampreys possess two intact *KCNA *tri-gene clusters, that would be robust support for the cluster duplication before the split between cyclostomes and gnathostomes. Addition of more sequences for different cyclostome lineages (e.g., southern hemisphere lampreys or hagfish) might also provide more evidence.

### Unique characters of sea lamprey genes: genome-wide phenomena?

We detected a peculiar amino acid composition in the sea lamprey *KCNA *genes (Figure 5A). Notably, this trend is not confined to the *KCNA *gene family: the peculiar protein-coding sequence properties, namely GC_3_, codon usage and amino acid composition seem to be genome-wide features (Figures 5B, 6D-E and 7).

In bacteria [18,25,26] and nematodes [20], the global GC-content is the major factor governing variations in amino acid composition of their genes. Based on our analysis, this appears to apply to vertebrates including the sea lamprey as well: the relative positions of species along the first factor of the correspondence analysis (CA) of amino acid composition were strongly correlated with their GC_3 _values rather than the evolutionary distances among them (Figure 5C). Other factors than GC-content apparently seem to contribute to the difference in amino acid compositions, since the first factor of the CA only accounted for ~63% of the total variance in amino acid composition (Figure 5B).

Sea lamprey genes show a strong correlation between codon usage bias, measured by ENc, and GC_3 _(Figure 6A), and its distribution suggests only weak selection on the codon usage in the sea lamprey genome (see Results). In contrast, GC_3 _only accounted for ~1/3 of total variance of codon usage pattern among sea lamprey genes (see Results), indicating that the codon usage patterns of sea lamprey genes are further affected by other factors. Variation of codon usage bias between vertebrate species was also mainly due to the GC-content as indicated by a strong correlation between ENc and GC_3 _and between the first factor of the CA of codon usage pattern and GC_3 _(Figure 6D and 6F). Above all, the sea lamprey exhibited an exceptionally high degree of codon usage bias among the vertebrates analyzed in the present study as it displayed the most extreme GC_3 _by far (Figure 6D). It is unknown whether the deviated amino acid composition and codon usage bias in the sea lamprey represents an ancient feature of the common vertebrate ancestor or is a derived feature acquired uniquely in the lamprey lineage. To address this question, it will be necessary to collect data from other lamprey species (e.g., southern hemisphere lampreys) and hagfishes.

### What is the biological significance of GC partitioning between coding and non-coding regions?

The peculiarity of the codon usage in the sea lamprey relative to that of other vertebrates draws a parallel in its variation among *Drosophila *species. An exceptionally low level of codon usage bias was observed in *Drosophila willistoni *compared to eleven other *Drosophila *species, which is related to the exceptionally low coding GC-content in this species [27]. Based on the estimate of the divergence time between *D. willistoni *and the *melanogster*/*obscura *groups, this change is estimated to have occurred within 45-30 million years [28]. In this case, changes in population size, mutational bias and tRNA abundance have been proposed as causal factors (discussed in [27]). These factors might also be associated with the peculiar codon usage in the sea lamprey.

One possible discrepancy in these biases between the sea lamprey and other vertebrates is the inherent nature of GC_3_. In vertebrates other than the lamprey, GC_3_, mentioned above as a source of biases in codon usage and amino acid composition (Figures 5C and 6D), is correlated with global genomic GC-content [29-32]. In lampreys, however, genomic sequences generally exhibit moderate GC-content of 40-50% [33], while GC-content in protein-coding regions, measured by GC_3 _(or GC_4_), is extremely high (70-90%) [34]. Similarly, genomic supercontigs containing sea lamprey *KCNA *genes identified in the present study had 41~51% of genomic GC-content (excluding repetitive elements), while GC_3 _of the *KCNA *genes ranged from 60 to 95%. The intra-genomic partitioning in GC-content between protein-coding and non-coding regions, seen also in the aforementioned *Drosophila *species, has not been reported before for any other vertebrate than the sea lamprey.

In principle, an employment of amino acid sequences in phylogenetic analyses is thought to buffer against the unfavorable effect caused by lineage-specific changes in GC-content and codon usage. However, our findings suggest that comparative sequence analyses with conventional methods involving lampreys at the amino acid level as well as at the nucleotide level can be misleading. The lineage-specific sequence properties that would affect most lamprey genes can cause the typically, but possibly incorrectly, recovered tree topology where lamprey sequences cluster at the basal branch of a vertebrate gene tree (Figure 1A). To accommodate the lineage-specific alteration of amino acid substitution models, we employed a phylogenetic tree inference method allowing a 'non-homogeneous' model [35,36]. This attempt resulted in a tree topology with more inconsistent relationships even inside gnathostome *KCNA *subgroups and did not lead to improvement for this particular gene family (data not shown). The possible causation between the lamprey lineage-specific sequence properties and their ambiguous phylogenetic positions should be verified with more sampling of gene families based on complete genomic sequences for this group of species.

## Conclusions

Our molecular phylogenetic analysis on the *KCNA *gene family resulted in low resolution of lamprey gene phylogeny. We identified lamprey's deviated amino acid composition and codon usage pattern from those of jawed vertebrates. It is possible that these are associated with the exceptionally high GC-content in protein-coding regions and frequently observed ambiguous molecular phylogeny of lamprey genes.

## Methods

### Sequences

Protein-coding nucleotide and amino acid sequences of human (*Homo sapiens*), mouse (*Mus musculus*), dog (*Canis familiaris*), opossum (*Monodelphis domestica*), platypus (*Ornithorhynchus anatinus*), chicken (*Gallus gallus*), anole lizard (*Anolis carolinensis*), western clawed frog (*Xenopus tropicalis*), zebrafish (*Danio rerio*), fugu (*Takifugu rubripes*), green spotted pufferfish (*Tetraodon nigroviridis*), medaka (*Oryzias latipes*) and three spine stickleback (*Gasterosteus aculeatus*) were downloaded from Ensembl version 53 [37] via BioMart. Nucleotide and amino acid sequences of the sea lamprey were downloaded from NCBI as GenBank flat files, and their protein-coding nucleotide sequences were extracted. Sequences that are derived from mitochondrial DNA or shorter than 100 amino acids were discarded. Partial genome assembly of the elephant shark was downloaded from http://esharkgenome.imcb.a-star.edu.sg/. Protein-coding sequences and their deduced amino acid sequences for this species were obtained by gene prediction using Genscan [38] with the organism setting 'Vertebrate'. Repetitive elements in the elephant shark genome were identified using RepeatModeler http://www.repeatmasker.org/RepeatModeler.html.

### *KCNA *sequence set

Sea lamprey genome assembly (PMAR3.0) was downloaded from the Genome Institute at Washington University ftp://genome.wustl.edu/pub/organism/Other_Vertebrates/Petromyzon_marinus/. We performed tBlastn using human KCNA protein sequences as queries against the sea lamprey genome assembly. All genomic segments with similarity (sequence alignment ≥ 25 amino acids, identity ≥ 30%) to the queries were retrieved and subjected to gene prediction with GeneWise [39] to extract coding nucleotide and amino acid sequences of *KCNA *and closely related genes outside the *KCNA *subfamily. To search *KCNA *genes of other species, amino acid sequences of human *KCNA *genes were used as queries to run Blastp searches against Ensembl peptides as well as the NCBI GenPept database. All hits with similarity (sequence alignment ≥ 25 amino acids, identity ≥ 30%) to human KCNA sequences were retrieved. To remove closely related homologs outside KCNA, all retrieved sequences were subjected to Blastp searches against human Ensembl peptide database (version 53), and only those with best hit to a KCNA were retained.

### Molecular phylogenetics

Multiple sequence alignments were constructed using MAFFT [40] with poorly aligned regions manually removed. The best models of amino acid substitutions were selected using ProtTest verstion 2.4 [41]. The LG+Γ_4 _model was selected as most suitable to the dataset for Figure S3, while the JTT+I+Γ_4 _was selected with the dataset for Figure 3. Maximum-likelihood trees were inferred using PhyML version 2.4.4 [42] under the selected substitution model. Support values for individual nodes were estimated with 1000 bootstrap replicates. Bayesian inference was performed using MrBayes.3.1.2 [43] under the selected model with four million generations (Additional file 2, Figure S2) and two million generations (Additional file 3, Figure S3) of two parallel runs. Consensus trees were generated after 'burn-in' for 25% sampled trees.

Phylogenetic positions of individual sea lamprey genes were further investigated by likelihood-based statistical tests under the assumed framework of gnathostome *KCNA *phylogeny documented previously (Figure 4) ([17]; also see Figure 2). The analysis was performed using Tree-Puzzle 5.2 [44] under the JTT+I+Γ_4 _model by inputting all possible tree topologies for relevant taxa in the 'user-defined tree' mode. For each tree, relationships within individual gnathostome *KCNA *subgroups were constrained according to the generally accepted species phylogeny. Relationships among *KCNA4, -7 *and *-1/2/3/5/6/10 *were constrained according to the previously supported phylogenetic scenario [17]. All three possible relationships between *KCNA1/2, 3/6 *and *5/10 *were regarded as equally likely. Under these conditions, we statistically analyzed positions of all seven sea lamprey *KCNA *genes individually by adding each of them to the gnathostome *KCNA *gene set. For each sea lamprey sequence, there were 45 possible tree topologies (Figure 4B). Probabilities of the tree topologies were calculated using CONSEL [45].

Probabilistic counts of gene duplications before (*N*_bef_) and after (*N*_aft_) the cyclostome-gnathostome split were performed as in a previous study [4]. For each sea lamprey *KCNA *gene, we divided all of the 45 possible tree topologies into three groups supporting scenarios I or/and II (see Figure 4A for details of scenario I and II). First, 21 tree topologies support scenario I in which the tri-gene cluster duplication occurred after the cyclostome-gnathostome split. Second, 18 tree topologies support scenario II in which the tri-gene cluster duplication occurred before the cyclostome-gnathostome split. Third, six tree topologies in which sea lamprey genes grouped with *KCNA4 *or *-7 *could not statistically distinguish between scenario I or II, In the third case, we assumed that scenario I and II are equally possible (Figure 4B).

### Correspondence analysis of amino acid composition

To investigate the amino acid composition, protein sequences for each species were concatenated, and frequencies of each amino acid were calculated. A correspondence analysis of amino acid composition was performed using the 'dudi.coa' function implemented in the ADE-4 package [46] under the R statistical computing environment. Only the longest peptide sequence per gene was used in this analysis. For the sea lamprey, to avoid biases in amino acid composition measurements caused by biased sampling of protein families, such as the abundant variable lymphocyte receptors [47], we removed identical or highly homologous sequences that show ≥ 60% similarity at the amino acid sequence level. Our final sequence set for the sea lamprey contained 173 genes (Additional file 5, Table S2). For the elephant shark, we used predicted peptide sequences mentioned above. To remove false positive gene predictions, all predicted peptide sequences were used as queries to run Blastp against protein sequences of human, chicken, western clawed frog, zebrafish, medaka and fugu, and only sequences with significant similarity (e-value ≤ 1e-20) were retained. The resulting sequences were further used as queries to run tBlastn against a repetitive element library built for this species (see above). All sequences with significant similarity (e-value ≤ 1e-20) to any repetitive element were discarded.

Amino acid composition of alignable regions of vertebrate *KCNA *proteins sequences was analyzed based on the aforementioned procedure. Partial *KCNA *sequences (containing < 70% of alignable region in length) were excluded. As variable regions account for substantial parts of KCNA proteins, a separate analysis was performed using the full-length protein sequences of the same set of *KCNA *genes.

### Synonymous codon usage analysis

Effective number of codons index (ENc) [21] and base compositions were calculated using codonW http://codonw.sourceforge.net/. Synonymous codon usage was investigated by within-group correspondence analysis (WCA) of codon counts [22,23] and by correspondence analysis of relative synonymous codon usage (CA-RSCU). The latter is the most widely used method for codon usage analysis, whereas the former has been recently demonstrated to produce more unbiased results because it takes into account both amino acid composition and codon degeneracy information [48]. For each species, protein-coding sequences were concatenated and frequencies of 59 synonymous codons were calculated. Correspondence analyses were performed using the 'dudi.coa' and 'within' functions in the ADE-4 package [46]. If a given gene had multiple alternative splicing variants, only the one with the longest coding sequence was used. Heat maps of relative synonymous codon usage were generated using CIMMiner http://discover.nci.nih.gov/cimminer.

## Authors' contributions

SK conceived the study. HQ and FH identified sea lamprey and elephant shark sequences. HQ analyzed molecular phylogeny, codon usage bias, GC-content and amino acid composition. SK and HQ wrote the first draft of the manuscript, and all authors contributed to the final version of the manuscript.

## Supplementary Material

Additional file 1 **Figure S1-Sequence alignment of three human KCNA and 16 new KCNA proteins that were newly identified in this study**. Only conserved regions are shown. Species names were abbreviated as in Figure 5. Click here for file

Additional file 2 **Figure S2-Maximum-likelihood tree of potassium voltage-gated channel, shaker-related (*KCNA*) genes rooted by other potassium channel protein families**. 253 amino acid sites were used in the analysis. Statistical support values for crucial nodes regarding the monophyly of the KCNA family are shown in order, bootstrap values in the ML analysis and posterior probabilities. A hyphen indicates inconsistent phylogenetic tree topology between Maximum-likelihood tree and Bayesian tree. Click here for file

Additional file 3 **Figure S3-Molecular phylogenetic tree of *KCNA *genes inferred with the Bayesian method**. Statistical support values for nodes with branches leading to sea lamprey or elephant shark genes are shown with ML bootstrap values left to the slash and posterior probabilities right to the slash. "-" indicates inconsistent topology between the ML tree and Bayesian tree. Click here for file

Additional file 4 **Table S1-All tree topologies within 1σ of log-likelihood from the ML trees for each sea lamprey *KCNA *gene**. Click here for file

Additional file 5 ****Table S2-Lamprey nucleotide sequences used in the present study****. Click here for file
