# Supplementary figures and images for: Unresolved orthology and peculiar coding sequence properties of lamprey genes: the KCNA gene family as test case
Source: BMC Genomics. 2011 Jun 23;12:325. doi: 10.1186/1471-2164-12-325 (PMC3141671; doi:10.1186/1471-2164-12-325)

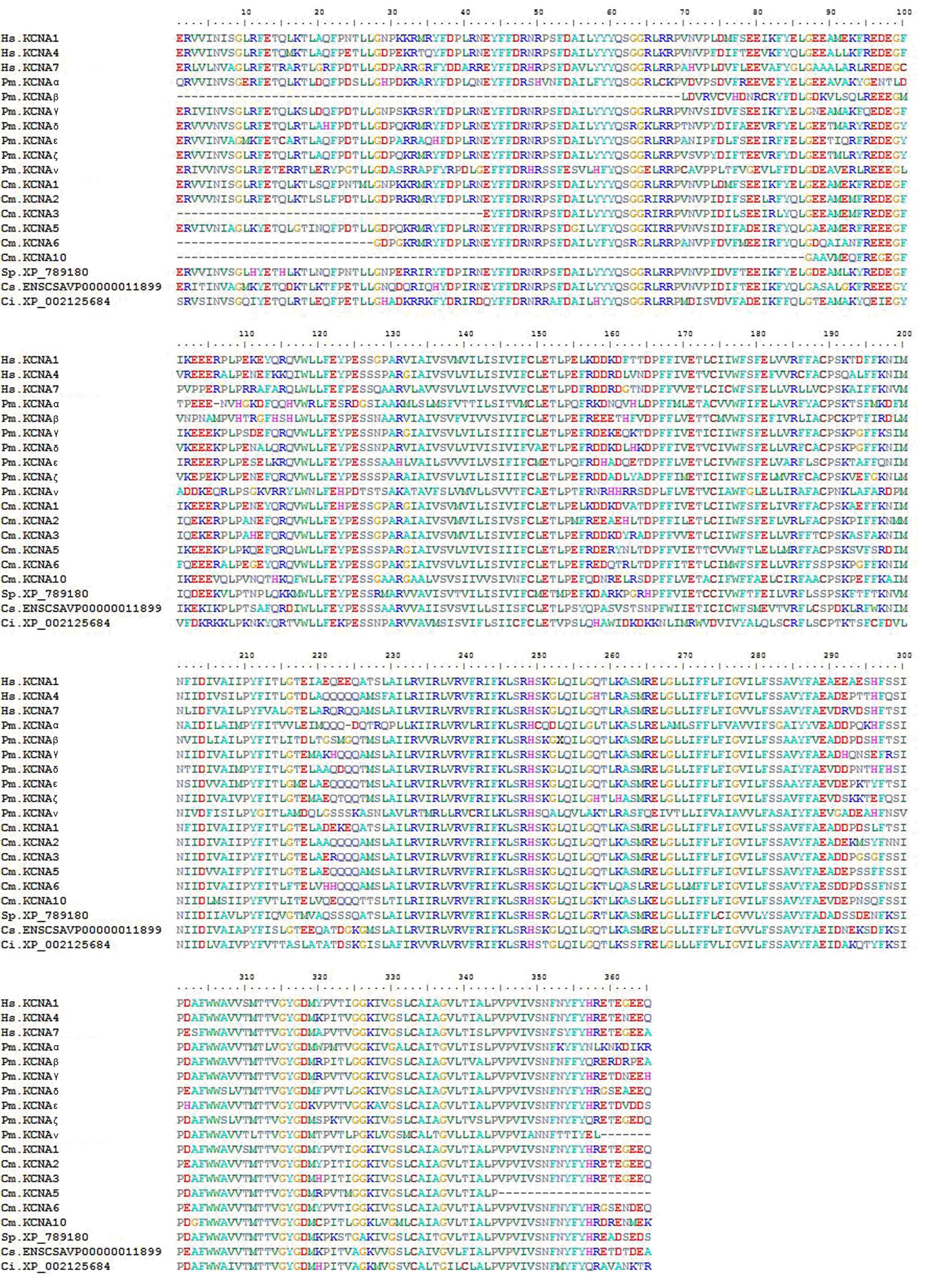

Supplement: Additional file 1 — Figure S1-Sequence alignment of three human KCNA and 16 new KCNA proteins that were newly identified in this study. Only conserved regions are shown. Species names were abbreviated as in Figure 5. [file 1471-2164-12-325-S1.JPEG]

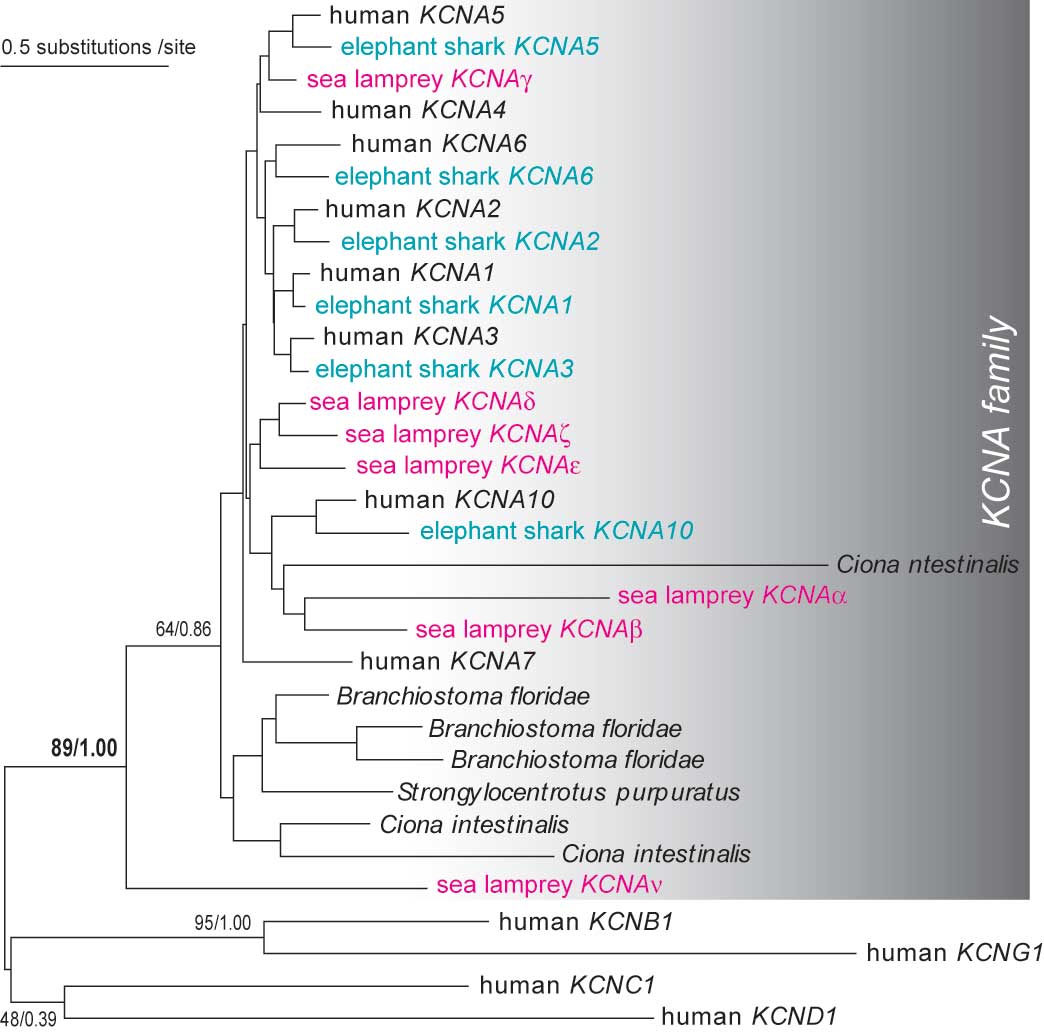

Supplement: Additional file 2 — Figure S2-Maximum-likelihood tree of potassium voltage-gated channel, shaker-related (KCNA) genes rooted by other potassium channel protein families. 253 amino acid sites were used in the analysis. Statistical support values for crucial nodes regarding the monophyly of the KCNA family are shown in order, bootstrap values in the ML analysis and posterior probabilities. A hyphen indicates inconsistent phylogenetic tree topology between Maximum-likelihood tree and Bayesian tree. [file 1471-2164-12-325-S2.JPEG]

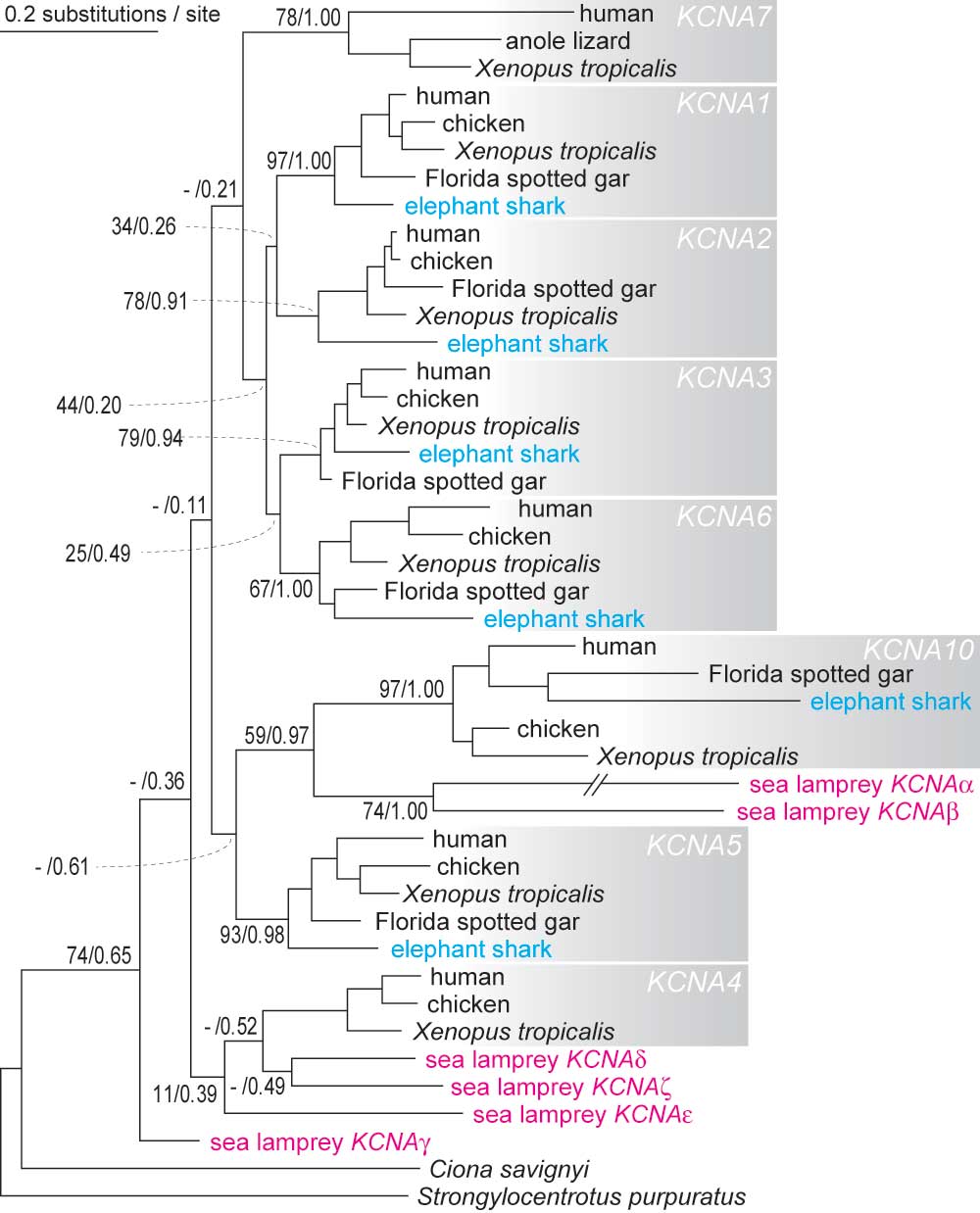

Supplement: Additional file 3 — Figure S3-Molecular phylogenetic tree of KCNA genes inferred with the Bayesian method. Statistical support values for nodes with branches leading to sea lamprey or elephant shark genes are shown with ML bootstrap values left to the slash and posterior probabilities right to the slash. "-" indicates inconsistent topology between the ML tree and Bayesian tree. [file 1471-2164-12-325-S3.JPEG]
