# Supplementary material for: Unresolved orthology and peculiar coding sequence properties of lamprey genes: the KCNA gene family as test case
Source: BMC Genomics. 2011 Jun 23;12:325. doi: 10.1186/1471-2164-12-325 (PMC3141671; doi:10.1186/1471-2164-12-325)
Supplement: Additional file 4 — Table S1-All tree topologies within 1σ of log-likelihood from the ML trees for each sea lamprey KCNA gene. [file 1471-2164-12-325-S4.DOC]

**Table S1. All tree topologies within 1σ of log-likelihood from the best trees for each sea lamprey *KCNA*** gene.

| Gene | Tree topology* | Supported scenario | log*L* | S.E. | ⊿log*L*  /S.E. | AU | BP | BPP | SH |
| --- | --- | --- | --- | --- | --- | --- | --- | --- | --- |
| *KCNAα* | ((((((1,2),(3,6)),(5,(10,p))),4),7),o) | II | -4337.12 | - | - | 0.91 | 0.71 | 0.96 | 0.98 |
|  | ((((((5,(10,p)),(3,6)),(1,2)),4),7),o) | II | -4340.61 | 4.47 | 0.78 | 0.36 | 0.19 | 0.03 | 0.61 |
| *KCNAδ* | ((((((1,2),(3,6)),(5,10)),(4,p)),7),o) | I/II | -4236.41 | - | - | 0.88 | 0.40 | 0.51 | 0.98 |
|  | (((((((1,2),(3,6)),(5,10)),4),p),7),o) | I | -4237.24 | 1.80 | 0.46 | 0.66 | 0.13 | 0.22 | 0.93 |
|  | (((((((1,2),(3,6)),(5,10)),p),4),7),o) | I | -4237.37 | 1.67 | 0.57 | 0.41 | 0.07 | 0.20 | 0.93 |
|  | ((((((1,2),(3,6)),(5,(10,p))),4),7),o) | II | -4239.80 | 4.98 | 0.68 | 0.43 | 0.18 | 0.02 | 0.68 |
| *KCNAζ* | ((((((1,2),(3,6)),(5,10)),(4,p)),7),o) | I/II | -4231.32 | - | - | 0.90 | 0.51 | 0.75 | 0.97 |
|  | ((((((1,2),(3,6)),(5,(10,p))),4),7),o) | II | -4234.65 | 4.83 | 0.69 | 0.42 | 0.12 | 0.03 | 0.71 |
|  | (((((((1,2),(3,6)),(5,10)),p),4),7),o) | I | -4233.48 | 3.01 | 0.71 | 0.50 | 0.06 | 0.09 | 0.85 |
|  | ((((((1,2),(3,6)),((5,p),10)),4),7),o) | II | -4234.75 | 4.78 | 0.72 | 0.41 | 0.11 | 0.02 | 0.71 |
|  | (((((((1,2),(3,6)),(5,10)),4),p),7),o) | I | -4233.56 | 2.88 | 0.78 | 0.33 | 0.05 | 0.08 | 0.83 |
|  | ((((((1,2),(3,6)),((5,10),p)),4),7),o) | I | -4235.47 | 4.21 | 0.99 | 0.14 | 0.01 | 0.01 | 0.64 |
|  | (((((((1,2),(3,6)),p),(5,10)),4),7),o) | I | -4235.48 | 4.2 | 0.99 | 0.10 | 0.01 | 0.01 | 0.64 |
| *KCNAβ* | ((((((1,2),(3,6)),((5,p),10)),4),7),o) | II | -4476.63 | - | - | 0.80 | 0.37 | 0.41 | 0.95 |
|  | ((((((1,2),(3,6)),(5,(10,p))),4),7),o) | II | -4477.45 | 2.77 | 0.30 | 0.58 | 0.25 | 0.18 | 0.83 |
|  | (((((((1,p),2),(3,6)),(5,10)),4),7),o) | II | -4478.41 | 2.95 | 0.60 | 0.43 | 0.12 | 0.07 | 0.76 |
|  | (((((((5,p),10),(3,6)),(1,2)),4),7),o) | II | -4480.24 | 4.52 | 0.80 | 0.39 | 0.10 | 0.01 | 0.51 |
|  | ((((((5,(10,p)),(3,6)),(1,2)),4),7),o) | II | -4481.17 | 5.22 | 0.87 | 0.28 | 0.05 | 0 | 0.38 |
|  | ((((((5,10),(3,6)),((1,p),2)),4),7),o) | II | -4481.82 | 5.68 | 0.91 | 0.31 | 0.05 | 0 | 0.31 |
| *KCNAγ* | (((((((1,2),(3,6)),(5,10)),4),p),7),o) | I | -4190.63 | - | - | 0.86 | 0.33 | 0.62 | 0.99 |
|  | (((((((1,2),(3,6)),(5,10)),4),7),p),o) | I | -4192.09 | 3.15 | 0.46 | 0.57 | 0.20 | 0.14 | 0.82 |
|  | ((((((1,2),(3,6)),((5,p),10)),4),7),o) | II | -4193.70 | 4.99 | 0.62 | 0.50 | 0.07 | 0.03 | 0.76 |
|  | (((((((1,2),(3,6)),p),(5,10)),4),7),o) | I | -4193.67 | 4.62 | 0.66 | 0.43 | 0.02 | 0.03 | 0.77 |
|  | ((((((1,2),(3,6)),((5,10),p)),4),7),o) | I | -4193.72 | 4.62 | 0.67 | 0.35 | 0.02 | 0.03 | 0.76 |
|  | (((((((1,2),(3,6)),(5,10)),p),4),7),o) | I | -4193.66 | 4.52 | 0.67 | 0.39 | 0.02 | 0.03 | 0.76 |
|  | ((((((1,2),(3,(6,p))),(5,10)),4),7),o) | II | -4195.28 | 6.5 | 0.72 | 0.43 | 0.06 | 0.01 | 0.64 |
|  | ((((((1,2),(3,6)),(5,(10,p))),4),7),o) | II | -4194.02 | 4.73 | 0.72 | 0.19 | 0.01 | 0.02 | 0.74 |
|  | ((((((1,2),((3,p),6)),(5,10)),4),7),o) | II | -4195.40 | 6.36 | 0.75 | 0.43 | 0.04 | 0.01 | 0.63 |
|  | ((((((1,2),(3,6)),(5,10)),(4,p)),7),o) | I/II | -4193.98 | 4.45 | 0.75 | 0.30 | 0.05 | 0.02 | 0.73 |
|  | ((((((1,2),((3,6),p)),(5,10)),4),7),o) | I | -4195.85 | 6.21 | 0.84 | 0.17 | 0.01 | 0 | 0.59 |
|  | ((((((5,10),(3,(6,p))),(1,2)),4),7),o) | II | -4198.42 | 8.62 | 0.90 | 0.33 | 0.04 | 0 | 0.33 |
|  | ((((((5,10),((3,p),6)),(1,2)),4),7),o) | II | -4198.45 | 8.59 | 0.91 | 0.33 | 0.04 | 0 | 0.33 |
|  | ((((((1,2),(3,6)),(5,10)),4),(7,p)),o) | I/II | -4193.09 | 2.64 | 0.93 | 0.17 | 0.03 | 0.05 | 0.74 |
|  | ((((((5,10),((3,6),p)),(1,2)),4),7),o) | I | -4198.91 | 8.42 | 0.98 | 0.12 | 0 | 0 | 0.30 |
| *KCNAν* | ((((((1,2),(3,6)),(5,10)),4),(7,p)),o) | I/II | -4486.30 | - | - | 0.72 | 0.33 | 0.21 | 0.91 |
|  | ((((((1,2),(3,6)),(5,10)),(4,p)),7),o) | I/II | -4486.76 | 2.97 | 0.15 | 0.61 | 0.24 | 0.13 | 0.85 |
|  | (((((((1,p),2),(3,6)),(5,10)),4),7),o) | II | -4487.02 | 2.29 | 0.31 | 0.56 | 0.11 | 0.10 | 0.89 |
|  | ((((((1,2),(3,(6,p))),(5,10)),4),7),o) | II | -4487.72 | 2.8 | 0.51 | 0.40 | 0.08 | 0.05 | 0.77 |
|  | ((((((1,2),(3,6)),((5,10),p)),4),7),o) | I | -4487.73 | 2.49 | 0.57 | 0.27 | 0 | 0.05 | 0.77 |
|  | (((((((1,2),(3,6)),p),(5,10)),4),7),o) | I | -4487.73 | 2.49 | 0.57 | 0.27 | 0 | 0.05 | 0.77 |
|  | (((((((1,2),(3,6)),(5,10)),p),4),7),o) | I | -4487.72 | 2.49 | 0.57 | 0.30 | 0 | 0.05 | 0.77 |
|  | (((((((1,2),(3,6)),(5,10)),4),p),7),o) | I | -4487.72 | 2.49 | 0.57 | 0.30 | 0 | 0.05 | 0.77 |
|  | ((((((1,2),(3,6)),(5,(10,p))),4),7),o) | II | -4487.76 | 2.55 | 0.57 | 0.27 | 0 | 0.05 | 0.78 |
|  | ((((((1,2),(3,6)),((5,p),10)),4),7),o) | II | -4487.80 | 2.53 | 0.59 | 0.21 | 0.01 | 0.05 | 0.77 |
|  | ((((((1,(2,p)),(3,6)),(5,10)),4),7),o) | II | -4487.86 | 2.55 | 0.61 | 0.23 | 0.01 | 0.04 | 0.77 |
|  | (((((((1,2),p),(3,6)),(5,10)),4),7),o) | I | -4487.97 | 2.51 | 0.67 | 0.25 | 0 | 0.04 | 0.75 |
|  | ((((((1,2),((3,6),p)),(5,10)),4),7),o) | I | -4488.07 | 2.51 | 0.71 | 0.03 | 0 | 0.04 | 0.73 |
|  | ((((((5,10),(3,6)),(1,2)),(4,p)),7),o) | I/II | -4490.58 | 5.58 | 0.77 | 0.31 | 0.05 | 0 | 0.38 |
|  | ((((((1,2),((3,p),6)),(5,10)),4),7),o) | II | -4488.26 | 2.54 | 0.77 | 0.09 | 0 | 0.03 | 0.70 |
|  | (((((((1,2),(3,6)),(5,10)),4),7),p),o) | I | -4488.08 | 2.26 | 0.79 | 0.06 | 0 | 0.04 | 0.71 |
|  | ((((((5,10),(3,6)),((1,p),2)),4),7),o) | II | -4490.59 | 5.35 | 0.8 | 0.32 | 0.04 | 0 | 0.36 |
|  | ((((((5,10),(3,6)),(1,2)),4),(7,p)),o) | I/II | -4490.11 | 4.68 | 0.81 | 0.31 | 0.07 | 0.01 | 0.44 |
|  | ((((((5,10),(3,(6,p))),(1,2)),4),7),o) | II | -4491.36 | 5.52 | 0.92 | 0.22 | 0.02 | 0 | 0.29 |
|  | ((((((5,10),(3,6)),(1,(2,p))),4),7),o) | II | -4491.46 | 5.41 | 0.95 | 0.16 | 0 | 0 | 0.27 |
|  | (((((((5,10),(3,6)),(1,2)),4),p),7),o) | I | -4491.50 | 5.35 | 0.97 | 0.14 | 0 | 0 | 0.26 |
|  | (((((((5,10),(3,6)),(1,2)),p),4),7),o) | I | -4491.50 | 5.35 | 0.97 | 0.13 | 0 | 0 | 0.26 |
|  | (((((((5,10),(3,6)),p),(1,2)),4),7),o) | I | -4491.57 | 5.41 | 0.97 | 0.13 | 0 | 0 | 0.26 |
|  | ((((((5,10),(3,6)),((1,2),p)),4),7),o) | I | -4491.57 | 5.41 | 0.97 | 0.14 | 0 | 0 | 0.26 |
| *KCNAε* | (((((((1,2),(3,6)),(5,10)),4),p),7),o) | I | -4268.43 | - | - | 0.73 | 0.12 | 0.13 | 0.98 |
|  | (((((((1,p),2),(3,6)),(5,10)),4),7),o) | II | -4268.59 | 4.77 | 0.03 | 0.63 | 0.08 | 0.11 | 0.95 |
|  | ((((((1,(2,p)),(3,6)),(5,10)),4),7),o) | II | -4268.59 | 4.77 | 0.03 | 0.63 | 0.04 | 0.11 | 0.95 |
|  | (((((((1,2),p),(3,6)),(5,10)),4),7),o) | I | -4268.59 | 4.77 | 0.03 | 0.63 | 0.02 | 0.11 | 0.95 |
|  | (((((((1,2),(3,6)),p),(5,10)),4),7),o) | I | -4268.58 | 4.46 | 0.03 | 0.68 | 0.09 | 0.11 | 0.97 |
|  | ((((((1,2),((3,6),p)),(5,10)),4),7),o) | I | -4268.87 | 4.89 | 0.09 | 0.47 | 0.06 | 0.08 | 0.93 |
|  | ((((((1,2),(3,6)),(5,(10,p))),4),7),o) | II | -4269.54 | 3.42 | 0.32 | 0.52 | 0.10 | 0.04 | 0.89 |
|  | ((((((1,2),((3,p),6)),(5,10)),4),7),o) | II | -4271.07 | 5.63 | 0.47 | 0.24 | 0.04 | 0.01 | 0.68 |
|  | ((((((1,2),(3,(6,p))),(5,10)),4),7),o) | II | -4271.07 | 5.63 | 0.47 | 0.24 | 0.04 | 0.01 | 0.68 |
|  | (((((((5,10),(3,6)),(1,2)),4),p),7),o) | I | -4270.63 | 4.32 | 0.51 | 0.56 | 0.06 | 0.02 | 0.79 |
|  | (((((((1,2),(3,6)),(5,10)),p),4),7),o) | I | -4269.94 | 2.98 | 0.51 | 0.44 | 0.02 | 0.03 | 0.92 |
|  | ((((((1,2),(3,6)),(5,10)),4),(7,p)),o) | I/II | -4269.18 | 1.41 | 0.53 | 0.46 | 0.05 | 0.06 | 0.89 |
|  | (((((((1,2),(3,6)),(5,10)),4),7),p),o) | I | -4269.18 | 1.41 | 0.53 | 0.46 | 0.05 | 0.06 | 0.89 |
|  | (((((((5,10),(3,6)),(1,2)),p),4),7),o) | I | -4271.49 | 5.33 | 0.57 | 0.46 | 0.03 | 0.01 | 0.74 |
|  | ((((((5,(10,p)),(3,6)),(1,2)),4),7),o) | II | -4271.72 | 5.69 | 0.58 | 0.43 | 0.05 | 0.01 | 0.66 |
|  | ((((((1,2),(3,6)),(5,10)),(4,p)),7),o) | I/II | -4270.16 | 2.76 | 0.63 | 0.36 | 0.03 | 0.02 | 0.86 |
|  | ((((((1,2),(3,6)),((5,10),p)),4),7),o) | I | -4270.69 | 3.54 | 0.64 | 0.17 | 0 | 0.01 | 0.85 |
|  | ((((((5,10),(3,6)),(1,2)),4),(7,p)),o) | I/II | -4271.54 | 4.69 | 0.66 | 0.23 | 0.02 | 0.01 | 0.65 |
|  | (((((((5,10),(3,6)),(1,2)),4),7),p),o) | I | -4271.54 | 4.69 | 0.66 | 0.33 | 0.05 | 0.01 | 0.65 |
|  | (((((((5,10),(3,6)),p),(1,2)),4),7),o) | I | -4272.22 | 5.67 | 0.67 | 0.23 | 0 | 0 | 0.64 |
|  | ((((((5,10),(3,6)),((1,p),2)),4),7),o) | II | -4272.22 | 5.67 | 0.67 | 0.23 | 0.01 | 0 | 0.64 |
|  | ((((((5,10),(3,6)),((1,2),p)),4),7),o) | I | -4272.22 | 5.67 | 0.67 | 0.23 | 0 | 0 | 0.64 |
|  | ((((((5,10),(3,6)),(1,(2,p))),4),7),o) | II | -4272.22 | 5.67 | 0.67 | 0.23 | 0 | 0 | 0.64 |
|  | ((((((5,10),(3,6)),(1,2)),(4,p)),7),o) | I/II | -4272.36 | 5.18 | 0.76 | 0.23 | 0.02 | 0 | 0.61 |
|  | ((((((5,10),((3,6),p)),(1,2)),4),7),o) | I | -4272.68 | 5.53 | 0.77 | 0.36 | 0 | 0 | 0.57 |
|  | (((((((5,10),p),(3,6)),(1,2)),4),7),o) | I | -4272.68 | 5.53 | 0.77 | 0.37 | 0 | 0 | 0.58 |
|  | ((((((1,2),(5,(10,p))),(3,6)),4),7),o) | II | -4272.84 | 5.52 | 0.80 | 0.11 | 0.02 | 0 | 0.55 |
|  | ((((((1,2),(3,6)),((5,p),10)),4),7),o) | II | -4271.25 | 3.37 | 0.84 | 0.1 | 0 | 0.01 | 0.76 |
|  | (((((((1,2),(5,10)),(3,6)),p),4),7),o) | I | -4272.72 | 5.07 | 0.85 | 0.12 | 0 | 0 | 0.60 |
|  | (((((((1,2),(5,10)),(3,6)),4),p),7),o) | I | -4271.85 | 3.9 | 0.88 | 0.19 | 0.01 | 0 | 0.67 |
|  | (((((((5,p),10),(3,6)),(1,2)),4),7),o) | II | -4273.46 | 5.63 | 0.89 | 0.10 | 0 | 0 | 0.47 |
|  | (((((((1,2),(5,10)),p),(3,6)),4),7),o) | I | -4273.46 | 5.44 | 0.93 | 0.02 | 0 | 0 | 0.48 |
|  | ((((((1,2),(5,10)),((3,6),p)),4),7),o) | I | -4273.46 | 5.44 | 0.93 | 0.03 | 0 | 0 | 0.48 |
|  | ((((((1,2),((5,10),p)),(3,6)),4),7),o) | I | -4273.46 | 5.44 | 0.93 | 0.02 | 0 | 0 | 0.48 |
|  | (((((((1,2),p),(5,10)),(3,6)),4),7),o) | I | -4273.37 | 5.32 | 0.93 | 0.03 | 0 | 0 | 0.50 |
|  | (((((((1,p),2),(5,10)),(3,6)),4),7),o) | II | -4273.37 | 5.32 | 0.93 | 0.02 | 0 | 0 | 0.50 |
|  | ((((((1,(2,p)),(5,10)),(3,6)),4),7),o) | II | -4273.37 | 5.32 | 0.93 | 0.03 | 0 | 0 | 0.50 |

The tree topologies were sorted by *Δ*log*L*/SE. Abbreviations: S.E., standard error of log*L*; BP, RELL BP; BPP, Bayesian posterior probability; AU, approximate unbiased test [1]; SH, Shimodaira-Hasegawa test [2]. *Abbreviations: *KCNA*: potassium voltage-gated channel, shaker-related gene; 1, *KCNA1*; 2, *KCNA2*; 3, *KCNA3*; 6, *KCNA6*; 5, *KCNA5*; 10, *KCNA10*; 4, *KCNA4*; 7, *KCNA7*; p, sea lamprey (*Petromyzon marinus*); o, outgroup.

1. Shimodaira H: **An approximately unbiased test of phylogenetic tree selection.** *Syst Biol* 2002, **51**:492-508.
2. Shimodaira, H, Hasegawa, M: **Multiple comparisons of log-likelihoods with applications to phylogenetic inference.** *Mol Biol Evol* 1999, **16**:1114–1116.
