# Supplementary material for: Unresolved orthology and peculiar coding sequence properties of lamprey genes: the KCNA gene family as test case
Source: BMC Genomics. 2011 Jun 23;12:325. doi: 10.1186/1471-2164-12-325 (PMC3141671; doi:10.1186/1471-2164-12-325)
Supplement: Additional file 5 — Table S2-Lamprey nucleotide sequences used in the present study. [file 1471-2164-12-325-S5.DOC]

**Table S2 -** Lamprey nucleotide sequences used in the present study.

| **No.** | **Genbank Accesion No** | **Gene Name** |
| --- | --- | --- |
| ***Petromyzon marinus*** | |  |
| 1 | AY750688 | testicular glycoprotein hormone receptor I precursor |
| 2 | DQ869188 | neurofilament subunit NF-L |
| 3 | DQ659108 | Tbx15/18 |
| 4 | DQ855960 | transthyretin (TTR) |
| 5 | DQ136025 | type II collagen alpha 1b (Col2a1b) |
| 6 | AY010116 | homeodomain protein DlxA (DlxA) |
| 7 | AY010118 | homeodomain protein DlxC (DlxC) |
| 8 | EF033074 | SPARCA (SPARC) |
| 9 | EF033076 | SPARCB (SPARC) |
| 10 | AY750689 | glycoprotein hormone receptor II |
| 11 | EF094822 | isolate PmCDA.21 cytosine deaminase |
| 12 | EF094823 | isolate PmCDA2 cytosine deaminase |
| 13 | EF094631 | isolate PmVLRA.1A03 variable lymphocyte receptor A diversity region (VLRA) |
| 14 | EF094660 | isolate PmVLRA.1C11 variable lymphocyte receptor A diversity region (VLRA) |
| 15 | EF094737 | isolate PmVLRA.36 variable lymphocyte receptor A diversity region (VLRA) |
| 16 | EF432251 | retinal photoreceptor cGMP-phosphodiesterase 6 catalytic subunit |
| 17 | EF166083 | gonadotropin-releasing hormone receptor 3 (gnrhr3) gene |
| 18 | AY028456 | estrogen receptor |
| 19 | AY028458 | progestin receptor |
| 20 | EF529216 | clone 4_LRRV variable lymphocyte receptor B cassette gene |
| 21 | EF529221 | clone 5_LRRV variable lymphocyte receptor B cassette gene |
| 22 | EF529223 | clone 8_LRRV variable lymphocyte receptor B cassette gene |
| 23 | D64055 | LMPX of lamprey |
| 24 | EF464184 | isolate PmVLRB.GS.C01 variable lymphocyte receptor diversity region gene |
| 25 | EF584739 | AMP-activated protein kinase gamma B |
| 26 | EF585497 | HVA calcium channel |
| 27 | EF585498 | small conductance calcium activated potassium channel |
| 28 | AF411465 | transcription factor Pax7 |
| 29 | AF411466 | transcription factor Pax2 |
| 30 | EU089674 | transcription factor AP-2 (AP-2) |
| 31 | EU089675 | Id (Id) |
| 32 | EU086587 | ZicA |
| 33 | EU086588 | FoxD-A |
| 34 | EU086590 | n-Myc |
| 35 | AM900682 | partial for putative ST8Sia IV alpha 28-sialyltransferase (siat8D gene) |
| 36 | AM900683 | partial for putative ST8Sia I alpha 28-sialyltransferase (siat8A gene) |
| 37 | AM900684 | partial for putative ST8Sia VI alpha 28-sialyltransferase (siat8F gene) |
| 38 | AF316877 | peroxisome proliferator-activated receptor |
| 39 | EU196399 | twist |
| 40 | EU196403 | Bapx1 |
| 41 | EU196409 | Gli1/2/3 |
| 42 | EU379655 | small GTPase Ras-dva gene |
| 43 | EU440327 | endothelin receptor A |
| 44 | EU449948 | repulsive guidance molecule (RGM) |
| 45 | EU449949 | neurotrophin |
| 46 | EU571207 | long photoreceptor transducin-alpha subunit |
| 47 | EU571209 | red-sensitive opsin |
| 48 | AF424736 | Ikaros-like transcription factor IKLF2 gene exons 6 7 and |
| 49 | AY077582 | vitellogenin-like protein |
| 50 | EU703786 | cyclooxygenase |
| 51 | FJ155927 | hypoxanthine-guanine phosphoribosyltransferase 1 (HPRT1) |
| 52 | M15891 | blood plasma apolipoprotein LAL1 |
| 53 | M15892 | blood plasma apolipoproitein LAL2 |
| 54 | M14773 | Lamprey (P.marinus) fibrinogen beta-chain |
| 55 | K03049 | Lamprey (Petromyzon marinus) fibrinogen gamma chain |
| 56 | M30123 | fibrinogen alpha chain |
| 57 | M74064 | lactate dehydrogenase (Ldh) |
| 58 | L05925 | Lamprey lamprin |
| 59 | M74193 | plasma albumin |
| 60 | FJ209300 | Trpc2 |
| 61 | FJ209301 | V1R vomeronasal receptor |
| 62 | FJ372630 | snail zinc finger protein (snail) |
| 63 | AJ458324 | paired superclass homeobox transcription factor (pitxa gene) |
| 64 | AF009964 | serpin |
| 65 | AB081461 | growth hormone |
| 66 | AB081462 | Insulin-like growth factor precursor |
| 67 | AF011898 | trypsinogen a2 (TRYPA2) |
| 68 | AY152675 | CD3 epsilon-associated signal transducer |
| 69 | AY152677 | clone 78D10 CD9-like protein |
| 70 | AY152678 | clone 87B05 CD9-like protein |
| 71 | AY152679 | CD98 solute carrier family 3 member 2 (SLC3A2) |
| 72 | AY152674 | phosphoinositide 3-kinase B-cell adaptor |
| 73 | AF031134 | serum albumin-like protein (AS) |
| 74 | AF032896 | polyadenylate binding protein (PABP) |
| 75 | AY171568 | ABCB9-like TAP-family protein (ABCB9) |
| 76 | AY178969 | chemokine receptor CXCR4 (CXCR4) |
| 77 | AY130349 | ribosomal protein L15 |
| 78 | AY130352 | ribosomal protein L17 |
| 79 | AY130357 | ribosomal protein L19 |
| 80 | AY130361 | ribosomal protein S2 |
| 81 | AY130391 | ferritin heavy chain polypeptide 1 |
| 82 | AY130394 | guanine nucleotide-binding protein |
| 83 | AY130398 | nucleoside diphosphate kinase |
| 84 | AY130400 | ribosomal protein L5 |
| 85 | AY130404 | ribosomal protein L7 |
| 86 | AY130408 | ribosomal protein L7A |
| 87 | AY130412 | ribosomal protein L9 |
| 88 | AY130416 | ribosomal protein L10 |
| 89 | AY130418 | ribosomal protein L11 |
| 90 | AY130426 | nuclear receptor subfamily 2 group F (NR2F2) |
| 91 | AY130430 | protein kinase C |
| 92 | AY130433 | protein tyrosine phosphatase non-receptor type 6 |
| 93 | AY130438 | ribosomal protein L8 |
| 94 | AY130447 | eukaryotic translation elongation factor 1 gamma |
| 95 | AY130450 | ribosomal protein S3 |
| 96 | AY130451 | ribosomal protein S5 |
| 97 | AY130452 | ribosomal protein S7 |
| 98 | AY130455 | ribosomal protein L18 |
| 99 | AY249863 | vitamin D receptor (VDR) |
| 100 | AY280622 | macrophage migration inhibitory factor (MIF) |
| 101 | AY333299 | isolate Pema-cathepsin cathepsin |
| 102 | AF099746 | Otx |
| 103 | AJ005434 | gene encoding dopamine D1 receptor partial |
| 104 | AJ005437 | gene encoding beta adrenoreceptor A partial |
| 105 | AJ005438 | gene encoding beta adrenoreceptor B partial |
| 106 | AY602220 | bone morphogenetic protein 24A (BMP24A) |
| 107 | AY602221 | bone morphogenetic protein 24B (BMP24B) |
| 108 | AY602222 | bone morphogenetic protein 24C (BMP24C) |
| 109 | AF129401 | engrailed-related homeobox protein (enga) gene exon 2 and |
| 110 | AY577947 | clone 1.4 variable lymphocyte receptor |
| 111 | AY578040 | clone 8.10C-10 variable lymphocyte receptor |
| 112 | AY578058 | clone PmGAPDH glyceraldehyde 3-phosphate dehydrogenase |
| 113 | AY577974 | clone 12.26 variable lymphocyte receptor gene |
| 114 | AY576797 | Vpre-B-like protein |
| 115 | AY576798 | V-like receptor |
| 116 | AY576799 | gicerin-like protein |
| 117 | AY576800 | nectin-like protein |
| 118 | AF108812 | homeobox protein SIX1 (Six1) |
| 119 | AY686861 | TCR-like |
| 120 | AY686862 | CD4-like |
| 121 | AY744918 | neogenin/DCC long isoform |
| 122 | AY744919 | UNC-5 |
| 123 | AY744920 | semaphorin 3 |
| 124 | AY744921 | semaphorin 4 |
| 125 | AY830453 | SoxE1 (SoxE1) |
| 126 | AY823514 | neuropeptide Y (NPY) |
| 127 | AY730276 | gonadotropin II beta subunit |
| 128 | U19361 | neurofilament subunit NF-180 |
| 129 | X59712 | CAAT-box DNA binding protein subunit B (NF-YB) |
| 130 | X64059 | partial for superoxide dismutase (Mn type) |
| 131 | AF159707 | proglucagon I precursor |
| 132 | AF159708 | proglucagon II precursor |
| 133 | DQ015877 | plexin A2 |
| 134 | DQ015878 | plexin A1 |
| 135 | AB205157 | CNP for C-type natriuretic peptide |
| 136 | DQ157849 | carbonic anhydrase |
| 137 | DQ151028 | clone 15.2 variable lymphocyte receptor diversity region (VLR) |
| 138 | DQ151105 | clone Ad.12 variable lymphocyte receptor diversity region (VLR) |
| 139 | DQ151113 | clone B.1 variable lymphocyte receptor diversity region (VLR) |
| 140 | DQ151235 | clone K.D8 variable lymphocyte receptor diversity region (VLR) |
| 141 | DQ151372 | clone T.G7 variable lymphocyte receptor diversity region (VLR) |
| 142 | AF248645 | hemoglobin PMII' |
| 143 | U20652 | scavenger receptor cyteine-rich domain superfamily Pema-SRCR |
| 144 | DQ275145 | sodium channel 2 |
| 145 | AF024595 | aryl hydrocarbon receptor (AHR) |
| 146 | DQ320318 | thyroid hormone receptor 2 |
| 147 | DQ320320 | retinoid X receptor 2 |
| 148 | DQ328984 | HMG box protein SoxE3 |
| 149 | AF247362 | Spi transcription factor |
| 150 | AB233469 | PQRFa for PQRFamide peptide |
| 151 | DQ370170 | patched |
| 152 | DQ370172 | clone 2 follistatin |
| 153 | DQ447196 | myosin VI (myo6) |
| 154 | DQ440978 | dystrophin |
| 155 | DQ457017 | gonadotropin releasing hormone II |
| 156 | AY152676 | CD45 protein tyrosine phosphatase receptor type C |
| 157 | EF094821 | variable lymphocyte receptor A (VLRA) gene |
| 158 | D55628 | POC for proopiocortin |
| 159 | AY577973 | clone 12.19 variable lymphocyte receptor gene |
| 160 | AY114108 | clone 7.2 hepatocyte nuclear factor 1 |
| 161 | AY168624 | TATA-binding protein (TBP) |
| 162 | AF424733 | Ikaros-like transcription factor IKLF1 |
| 163 | AF434665 | Hox1w (hox1w) |
| 164 | AY028457 | corticoid receptor |
| 165 | AF247828 | biglycan-like protein 2 (BGL2) |
| 166 | EF529284 | clone 5 variable lymphocyte receptor B cassette gene |
| 167 | AY861455 | retinoic acid receptor |
| 168 | M84482 | (lamprey) fibrinogen alpha-2 chain 3'end |
| 169 | EF529218 | clone 4 variable lymphocyte receptor B cassette gene |
| 170 | D55629 | proopiomelanotropin |
| 171 | EF166082 | gonadotropin-releasing hormone receptor 2 (gnrhr2) gene |
| 172 | EF528625 | clone LRRN_LRR1 variable lymphocyte receptor A cassette gene |
| 173 | AF439802 | putative gonadotropin releasing-hormone receptor |
